# Surviving Starvation: Proteomic and Lipidomic Profiling of Nutrient Deprivation in the Smallest Known Free-Living Eukaryote

**DOI:** 10.3390/metabo10070273

**Published:** 2020-07-03

**Authors:** Sarah F. Martin, Mary K. Doherty, Eliane Salvo-Chirnside, Seshu R. Tammireddy, Jiaxiuyu Liu, Thierry Le Bihan, Phillip D. Whitfield

**Affiliations:** 1 Kinetic Parameter Facility, SynthSys—Synthetic and Systems Biology, The University of Edinburgh, Edinburgh EH9 3BD, UK; sarah.martin@gov.scot (S.F.M.); eliane.chirnside@ed.ac.uk (E.S.-C.); jiaxiuyu.liu@ed.ac.uk (J.L.); 2 Lipidomics Research Facility, University of the Highlands and Islands, Inverness IV2 3JH, UK; mary.doherty@uhi.ac.uk (M.K.D.); seshu.tammireddy@uhi.ac.uk (S.R.T.)

**Keywords:** algae, lipid metabolism, nitrogen, *Ostreococcus tauri*, phosphorus, proteome

## Abstract

Marine phytoplankton, comprising cyanobacteria, micro- and pico-algae are key to photosynthesis, oxygen production and carbon assimilation on Earth. The unicellular green picoalga *Ostreococcus tauri* holds a key position at the base of the green lineage of plants, which makes it an interesting model organism. *O. tauri* has adapted to survive in low levels of nitrogen and phosphorus in the open ocean and also during rapid changes in the levels of these nutrients in coastal waters. In this study, we have employed untargeted proteomic and lipidomic strategies to investigate the molecular responses of *O. tauri* to low-nitrogen and low-phosphorus environments. In the absence of external nitrogen, there was an elevation in the expression of ammonia and urea transporter proteins together with an accumulation of triglycerides. In phosphate-limiting conditions, the expression levels of phosphokinases and phosphate transporters were increased, indicating an attempt to maximise scavenging opportunities as opposed to energy conservation conditions. The production of betaine lipids was also elevated, highlighting a shift away from phospholipid metabolism. This finding was supported by the putative identification of betaine synthase in *O. tauri*. This work offers additional perspectives on the complex strategies that underpin the adaptive processes of the smallest known free-living eukaryote to alterations in environmental conditions.

## 1. Introduction

Marine phytoplankton, comprising cyanobacteria, micro- and pico-algae are responsible for approximately 50% of photosynthesis, oxygen production and carbon assimilation on Earth while making up less than 1% of global biomass [1]. Vast areas of open ocean show little or no chlorophyll presence, as primary productivity is concentrated in nutrient-rich coastal areas and oxygen-rich polar oceans supported by upwelling of deeper water [2,3]. 

The unicellular pico-alga, *Ostreococcus tauri,* is the smallest known free-living eukaryote and holds a key position at the base of the green lineage of plants, which makes it an interesting and increasingly well-described model organism. Discovered in 1994 in the Thau lagoon in France [4], *O. tauri* has been isolated in oceans and coastal regions across the globe. *O. tauri* exhibits one of the simplest ultrastructures, lacking a structured cell wall and containing only a single chloroplast, mitochondrion, Golgi body and nucleus [5]. Its genome is also very compact with 8200 genes across 12.6 MB [6]. Several ecotypes of *Ostreococcus* have been characterised, including high-nutrient, high-light coastal strains as well as open-ocean strains, which grow in deeper waters near the critical photosynthetic depth in gyre zones [7]. Like many phytoplankton, *O. tauri* survives under a range of environmental conditions and the availability of primary nutrient sources is one of the main factors that influence its distribution. 

Nitrogen in the form of nitrate (NO_3_) and phosphorus as inorganic phosphate (PO_4_) are the two limiting phytoplankton macro-nutrients, as evidenced by their exhaustion in productive surface waters [8]. These nutrients can change both seasonally and spatially [9], nitrate commonly during summer months and phosphate typically in the spring and at fresh-to-saline water transitions [10]. The oceanic sources and bioavailability of nitrate and phosphate have been studied extensively, as have their effects on the biological carbon pump and their impact on atmospheric CO_2_ [11,12,13]. The oceanic nitrogen:phosphorus ratio is tightly balanced at the Redfield ratio 16:1 [14] by nitrification in the presence of sufficient phosphate and oxygen by atmospheric nitrogen-fixing prokaryotes, and by denitrification in anaerobic, phosphate-limited conditions [15]. However, unlike nitrogen, phosphate enters oceans solely through weathered minerals from rivers, and is hence critical to productivity not only during short-term stratification, but also on geological timescales [16]. 

The oversupply of nitrate and phosphate due to agricultural fertilizers, human waste, manure and the loss of restoring wetlands does not eliminate nutrient limitation, as algal blooms will rapidly expand to scavenge these nutrient-rich environments, generating hypoxic, eutrophic and even toxic environments in their wake [17]. Excessive concentrations are therefore considered environmental pollutants. Even in productive waters, the availability of nutrients fluctuates and phytoplankton have adapted to survive, if not prosper, in both low levels of nutrients in the open ocean, and also during rapid changes in relative levels of nitrogen and phosphate sources in coastal waters.

The deprivation of nitrate and phosphate is known to mediate strong effects on lipid metabolism in microalgae [18,19]. Nitrate limitation leads to the accumulation of triacylglycerols [20], whereas phosphate depletion results in an increase in non-phosphorous, betaine lipids [21,22]. This switch in their metabolism permits algae to adjust to environmental changes, a response that has been exploited by the biofuel and biotechnology industries [23,24]. Researchers have increasingly utilised advanced analytical approaches to determine the molecular adaptations at the lipid level associated with nutrient stress. Lipid remodelling has been investigated in a variety of microalgae including *Chlorella sp.* [25,26], *Nannochloropsis sp.* [27], *Chlamydomonas reinhardtii* [28], *Phaeodactylum tricornutum* [29], *Ettlia oleoabundans* [30], *Emiliania huxleyi* [31] and *O. tauri.* [32].

In the current study, we have extended this work and describe a system-scale investigation of nitrogen and phosphorus deprivation in the common global picoplankton *O. tauri* that combines untargeted proteomic and lipidomic strategies. The profiling of proteins and lipids captures cellular activity by identifying and quantifying products of gene expression and metabolism, thus providing proof of the implementation of putative adaptation strategies inferred from the genome. As such, the ability to obtain multilevel evidence of strategies to overcome nutrient limitation may be crucial for understanding adaptive processes of phytoplankton during short- and long-term shifts in biogeochemical processes in the oceans.

## 2. Results and Discussion

### 2.1. Temporal Profiling of O. tauri Proteome under Nutrient Stress

*O. tauri* cultures were monitored and sampled for three days after switching cells to media lacking nitrogen sources (N-dep), media lacking phosphate (P-dep), media lacking both nutrients and complete control media. The optical density of cultures increased from 0.12 ± 0.01 at the start of the time course to 0.31 ± 0.02 (N-dep cultures) and 0.38 ± 0.03 (control and P-dep cultures), corresponding to an approximate tripling of cell numbers in all cultures over three days. Cultures were sampled during linear growth and none of the cultures plateaued within the three monitored days. Interestingly, the P-dep culture showed no slowing of growth. The relative effect of nitrogen and phosphorus supply (and limitation) on algal growth has been an area of interest for algal biotechnologists for many years. Our data would support the observation by Fong et al. [33] that algal biomass is not directly controlled by P supply, particularly for green algae. A caveat to that assertion is that the present study is relatively short compared to field studies. A shotgun proteomic analysis was subsequently performed. Across the time course for all conditions, a total of 1245 proteins were identified, of which 761 were identified and quantified with at least two peptides (Table S1). 

**Figure 1:**
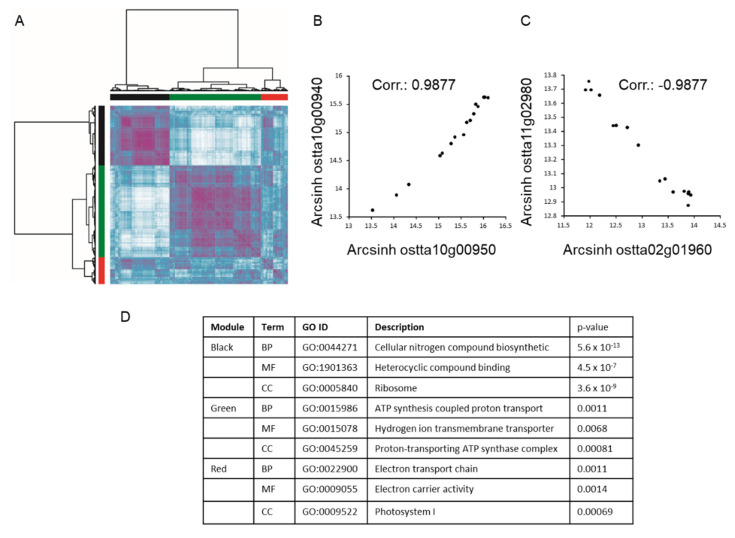
Clustering of the proteomic label-free analysis of *O. tauri*. *O. tauri* were sampled at *t* = 0, 24, 48 and 72 h in complete media, N-dep, P-dep and NP-dep conditions. (**A**) The data showed three major clusters. The clustering heat map was created using no threshold. The scale ranges from white to purple, with white associated to anti-correlated protein pair and purple representing high correlation between protein pair. (**B**) High correlation between co-expressed protein ostta10g00940 (Nar2) and ostta10g00950 (Ntr), two nitrogen protein transporters. (**C**) An example of two highly anti-correlated proteins—ostta11g02980, a N6-DNA-methyltransferase, and ostta02g01960, Btf3 BTF3 transcription factor. (**D**) GO term enrichment analysis of the three main cluster identified in (**A**) using topGO.

Nutrient stress imposes important changes at the proteome level, which are tightly regulated and co-ordinated. We used a protein co-expression analysis to highlight the correlation between proteins under the different nutrient conditions as illustrated in Figure 1A. Three major protein clusters emerged—“Black”, “Green” and “Red” clusters. The Black cluster shows a high level of correlation between the different proteins composing this cluster (purple); a similar finding is also observed with the Green cluster. These two clusters, however, are anti-correlated (white region off-diagonal from Figure 1A). A GO enrichment term analysis using topGO [34] was performed, which showed a significant enrichment term for the Black cluster, associated with nitrogen synthesis, whilst the Green cluster (slightly less enriched) is associated with ATP synthesis coupled proton transport (Figure 1D). As illustrated in Figure 1B, a similar pattern of expression is observed between Nar2 (ostta10g00940) and Ntr2 (ostta10g00950), which are part of a nitrogen transporter protein cluster coded on chromosome 10. The co-expression of these two proteins is clearly highlighted in Figure 1B with a coefficient correlation of 0.9877 observed between those two proteins. In contrast, among all the proteins that were anti-correlated, ostta11g02980, a predicted N6-DNA-methyltransferase, and ostta02g01960, a BTF3 transcription factor, were found to be highly anti-correlated (Figure 1C). 

**Figure 2:**
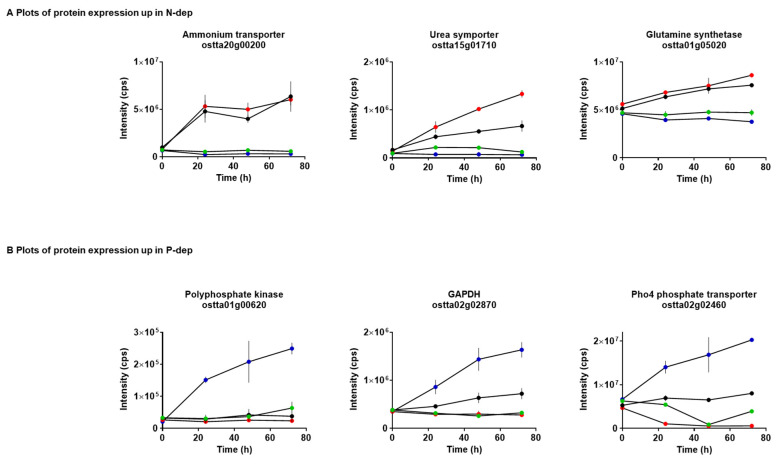
Temporal increases in protein expression in *O. tauri* cultured under N-dep or P-dep conditions. The expression of proteins associated with (**A**) nitrogen metabolism and (**B**) phosphate scavenging was elevated over 72 h. Key: Complete media = green; N-dep = red; P-dep = blue; NP-dep = black.

The expression of a number of proteins involved in nitrogen assimilation and nitrate metabolism was shown to be significantly changed under nitrogen-depleted conditions (Figure 2A). This included the upregulation of the ammonium transporter (ostta20g00200), urea high-affinity symporter DUR 3 (ostta15g01710) and ATP-dependent glutamine synthetase (ostta01g05020), which catalyses the incorporation of inorganic ammonium into glutamine. These findings are in agreement with studies of other eukaryotic microalgae where nitrogen limitation has been shown to lead to changes in the expression of assimilation proteins and scavenging of alternative nitrogen sources [35,36,37]. The response of *O. tauri* to N-dep conditions may highlight a key survival adaptation for viability in low inorganic nitrogen environments. 

P-dep also led to the differential expression of a number of proteins in *O. tauri* across the 72 h period (Figure 2B). Of particular note were polyphosphate kinase (ostta01g00620), GAPDH (ostta02g02870) and Pho4 (ostta02g02460). Pho4 is a Na^+^-coupled high-affinity phosphate transporter. Expression of related genes in *Saccharomyces scerevisiae* and *Neurospora crassa* [38] is highly correlated with the external Na^+^ and inorganic phosphate concentration. Phosphorus plays a regulatory role in Rubisco activation, phosphorylation of intermediates of the Calvin cycle, as well as energy availability [39]. Phylogenetic analysis of these proteins [40] revealed orthologues in *Chlorophyta* and *Viridiplantae* and, excluding polyphosphate kinase, with wider *Eukaryota* (Figure S1). The level of orthology varies between the different proteins, with between 3 and 6 orthologs of each observed at the *Mamiellales* level rising to between 28 (polyphosphate kinase) and over 5000 (ammonium transporter) at the *Eukaryota* level.

The results here are consistent with strategies employed by phytoplankton for survival in low-phosphate environments—upregulation of enzymes to hydrolyse dissolved organic phosphate (e.g., phosphomonoesters and phosphonates) into phosphate [41,42] and induction of high-affinity phosphate scavenging systems. Indeed, high-affinity phosphate transport may be a key adaptation of marine phytoplankton that enables them to persist in low-phosphorus conditions [39].

**Figure 3:**
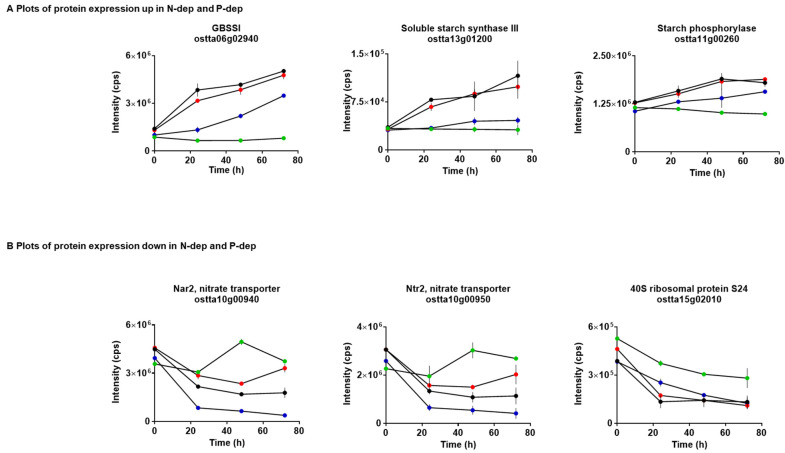
Differential expression of proteins in both N-dep and P-dep conditions. A number of proteins showed (**A**) increased and (**B**) decreased expression in response to the different nutrient-limiting conditions. Key: Complete media = green; N-dep = red; P-dep = blue; NP-dep = black.

Under nutrient-replete conditions, the starch granule is a key energy store for *O. tauri*. A number of proteins involved in starch biosynthesis showed altered profiles of expression as a result of nutrient deprivation (Figure 3A). The abundance of granule-bound starch synthase (GBSSI) (ostta06g02940) was significantly higher in N-dep, P-dep and NP-dep than *O. tauri* grown in complete media. Interestingly, an elevated level of expression of GBSSI was observed in N-dep and NP-dep compared to P-dep. The expression of soluble starch synthase III (SSIII) (ostta13g01200) was also increased, although this was more pronounced in N-dep and NP-dep, suggesting divergent regulation of these proteins under nitrogen and phosphate deprivation.

These two enzymes regulate the production of glucans bound in the starch granule (specifically, the synthesis of amylose through extension of amylopectin chains) and have been shown to increase starch synthesis during light hours to peak storage at night under circadian control [43]. With nutrient limitation, starch synthesis appears to be an initial global response, which may prolong survival under low nutrient availability. In tomato plants, nitrogen limitation increases foliar starch levels and decreases oxygen sensitivity of CO_2_ fixation, which in turn leads to further increases in starch levels and a limitation of photosynthesis [44]. In plants, the rate of net CO_2_ fixation has been reported to increase with decreasing leaf nitrogen concentration [45]. Phosphate deprivation, however, results in an increase in oxygen sensitivity of CO_2_ fixation, decreasing starch synthesis. Another enzyme, starch phosphorylase (ostta11g00260), was also significantly upregulated. This protein catalyses the reversible transfer of glucosyl units to alpha-1,4-D-glucan chains with the release of phosphate, indicating increased phosphorolytic degradation of starch as well as synthesis in nutrient limiting conditions.

In contrast, ribosomal proteins were downregulated in N-dep, P-dep and NP-dep conditions (Figure 3B). This suggests that there may be a reduction in the biosynthesis of proteins, which reflects a general stress response. Similarly, the expression of two nitrate transporters, Nar2 (ostta10g00940) and Ntr2 (ostta10g00950), was significantly decreased within 72 h in both nitrogen- and phosphorus-deficient media. The highest concentration of inorganic nitrogen in oceans is in the form of nitrate. During uptake, phytoplankton reduce nitrate to ammonium through the rate-limiting enzyme nitrate reductase (Nia) [46]. The expression of this protein in *O. tauri* (ostta10g00920) was also observed to be downregulated in all of the nutrient-limiting conditions. It is crucial that algae regulate the process of nitrate reduction, as it is a highly energy-intensive process. Furthermore, the intermediate product of nitrate reduction, nitrite, is cytotoxic and mutagenic. Increasing concentrations of ammonia (NH_4_) have been shown to inhibit nitrate uptake through inhibition of nitrate reductase [47,48]. In low-nitrogen open ocean environments, recycled ammonium drives production. Ammonia does not require the reduction step and is taken up through the ATP-dependent glutamine synthetase (up in N-dep, also Dur3) and an ammonium transporter [49]. 

### 2.2. Fractionation Provides In-Depth Characterisation of Differentially Expressed O. tauri Proteins Induced by Nutrient Depletion

It is important to note that a significant proportion of identified proteins were of unknown function in an otherwise well-annotated genome, pointing towards further work on a number of mechanisms in *O. tauri* that are distinct from sequenced and studied species. In order to explore the proteome of *O. tauri* in greater depth, the soluble protein complement from complete media, N-dep and P-dep cultures harvested at 48 h were subjected to isoelectric focusing (IEF). A total of 2021 proteins and 1140 proteins using two or more peptides were identified. This represents 11% coverage of the theoretically expressed proteome of *O. tauri* and 1.5 times more than the non-fractionated approach presented above. Of these proteins, 144 were significantly different between nitrogen/phosphorus-depleted and replete conditions (Table S2). There was an overall upregulation of proteins in N-dep and P-dep conditions, and 25% of these proteins were found to be upregulated in both nitrogen- and phosphorus-limiting conditions. These results demonstrate the resolving power of this fractionation strategy that permits more extensive coverage of the *O. tauri* proteome, although this form of analysis extends sample preparation and requires additional liquid chromatography mass spectrometry (LC-MS) run time.

Nitrogen depletion leads to the accumulation of neutral storage lipids in algae, mostly triacylglycerols (TG). From the IEF fractionated data sets, we examined the proteome of *O. tauri* for the enzymes involved in TG biosynthesis. A number of the enzymes were identified, including glycerol phosphate acyltransferase (GPAT) (ostta02g00340) and 1-acyl-sn-glycerol-3-phosphate acyltransferase (LPAT) (ostta07g03340), although the expression levels of these proteins were not consistently upregulated under N-dep conditions. This finding may reflect the temporal lag between gene expression and lipid accumulation in *O. tauri*, or could indicate that by 48 h, the algae have reached a steady state when nitrogen is limiting. 

Similarly, phytoplankton have the ability to alter lipid metabolism in response to phosphorus deprivation. In *O. tauri* cultured under P-dep conditions, a protein was observed, which, from the genome sequence, was attributable to a hypothetical protein (ostta15g02860). A subsequent BLAST search was performed and the domains DUF3419 superfamily and AdoMet-Mtases superfamily were putatively identified. These domains are homologous to betaine lipid synthase (BTA1) from *Chlamydomonas reinhardtii* [50]. BTA1 catalyses the biosynthesis of diacylglyceryl-*N*,*N*,*N*-trimethylhomoserine (DGTS) and, to the authors’ knowledge, this is the first report of this enzyme in *O. tauri*. In other algal species, it has also been established that DGTS can act as a precursor for the related betaine lipid diacylglyceryl-hydroxy-methyl-*N*,*N*,*N*-trimethyl-β-alanine (DGTA) [51].

### 2.3. Lipid Profiling of O. tauri under Nitrogen- and Phosphorus-Limiting Conditions

**Figure 4:**
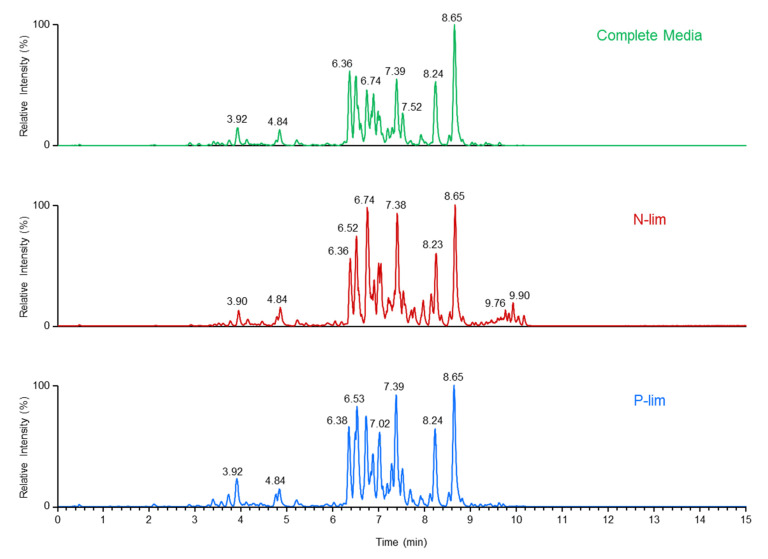
Representative LC-MS chromatograms of lipid profiles of *O. tauri*. Lipids were analysed in positive ion mode. Highly reproducible elution profiles were observed for algae grown in the different culturing conditions. The lipidomic analysis revealed the presence of a variety of polar and non-polar lipids.

We also conducted a parallel study on the 48 h cultures to determine the lipid changes in *O. tauri* triggered by nutrient depletion using high-resolution LC-MS. This direct profiling approach avoided the need for laborious, multistep processes employed in conventional methodologies that involve isolation and purification of algal lipids prior to their analysis. Representative positive ion LC-MS chromatograms of algae grown in N-dep, P-dep and complete media are shown in Figure 4 (for negative ion mode data, see Figure S1). In agreement with the elegant study by Degraeve-Guilbault et al. [32] we detected a variety of lipids in *O. tauri*, including molecular species of phosphatidylglycerol (PG), phosphatidylethanolamine (PE), monogalactosyl diacylglycerol (MGDG), digalactosyl diacylglycerol (DGDG) and sulfoquinovosyl diacylglycerol (SQDG), although there was a complete absence of phosphatidylcholine (PC), even in cultures grown under replete conditions. Our LC-MS analysis also identified the sulphur-containing phospholipid, phosphatidyldimethylpropanethiol (PDPT). Of particular note were intense ion signals in positive ion mode at mass-to-charge ratios (*m*/*z*) of 715.4373, 743.4696, 795.5004 and 823.5318 that were attributable to the protonated ions of PDPT 30:4, 32:4, 36:6 and 38:6, respectively [32,52]. 

**Figure 5:**
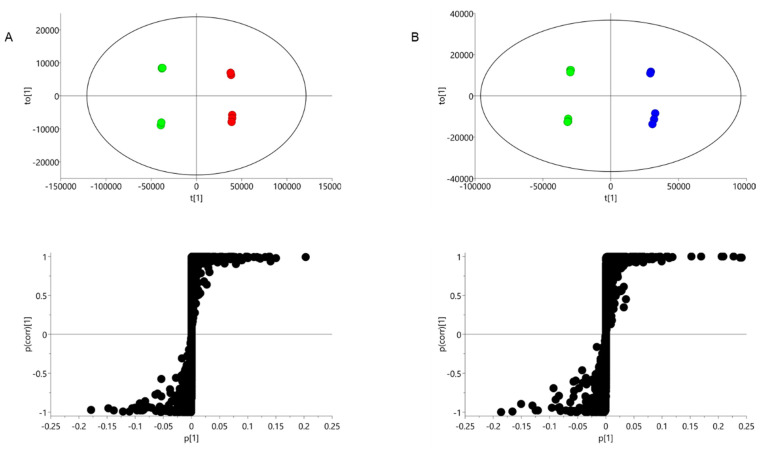
Characterising the effects of nutrient deprivation on lipid metabolism. OPLS-DA scores plots and associated S-plot of positive ion data characterising the disturbances in lipid metabolism in *O. tauri* induced by (**A**) N-dep and (**B**) P-dep conditions. Key: Complete media = green; N-dep = red; P-dep = blue.

In order to characterise the metabolic changes induced by the nutrient-limiting conditions, the data sets were subjected to multivariate data analysis. Principal Component Analysis (PCA) effectively discriminated the complete media, N-dep and P-dep groups, indicating that there were differences in their lipid composition (Figure S2). Having established clustering behaviour, the lipidomic data sets were further interrogated by Orthogonal Projections to Latent Structures Discriminant Analysis (OPLS-DA). The OPLS-DA models of the positive ion data were able to distinguish the cultures grown in complete media from those subjected to either N-dep or P-dep environments (Figure 5A,B and Table S3). The biochemical differences in *O. tauri* resulting from nutrient stress are represented in regions away from the algae culture in complete media. In comparison, the lipid changes with the negative ion data were not as pronounced (Figures S3 and S4 and Table S4).

The associated S-plots of the positive ion data permitted clear visualisation of the alterations of lipid composition. The lipids that showed the maximum change are plotted at the top or bottom of the S-plot. Analysis revealed that in *O. tauri* cultured in N-dep media, there was a substantial accumulation of neutral lipids in the form of molecular species of TG (Table S3). The increase in levels range from 1.5- (TG 32:6) to 20-fold (TG 54:6). Similarly, there was elevation in diacylglycerol (DG) species compared to algae grown in complete media (Table S3). 

In P-dep conditions, there was also a modest elevation of TG and DG species. These changes were accompanied by an increased abundance of betaine lipids (Table S3), which are major membrane components in algae. The presence of DGTA lipid diacylglyceryl-hydroxy-methyl-*N*,*N*,*N*-trimethyl-β-alanine (DGTA) has previously been reported to be the exclusive betaine lipid in *O. tauri* [30]. As a result, in this study, betaine lipids have been nominally assigned as DGTA. There were also committant decreases in phospholipids, specifically, molecular species of phosphatidylglycerol (PG) and PDPT. Other phospholipids classes such as phosphatidylinositol (PI) were not found in high abundance. These findings indicate that a metabolic shift towards non-phosphorous lipids is a strategy adopted by *O. tauri* for the conservation of phosphorous in P-dep environments. 

## 3. Materials and Methods 

### 3.1. Culturing and Harvesting of O. tauri

**Figure 6:**
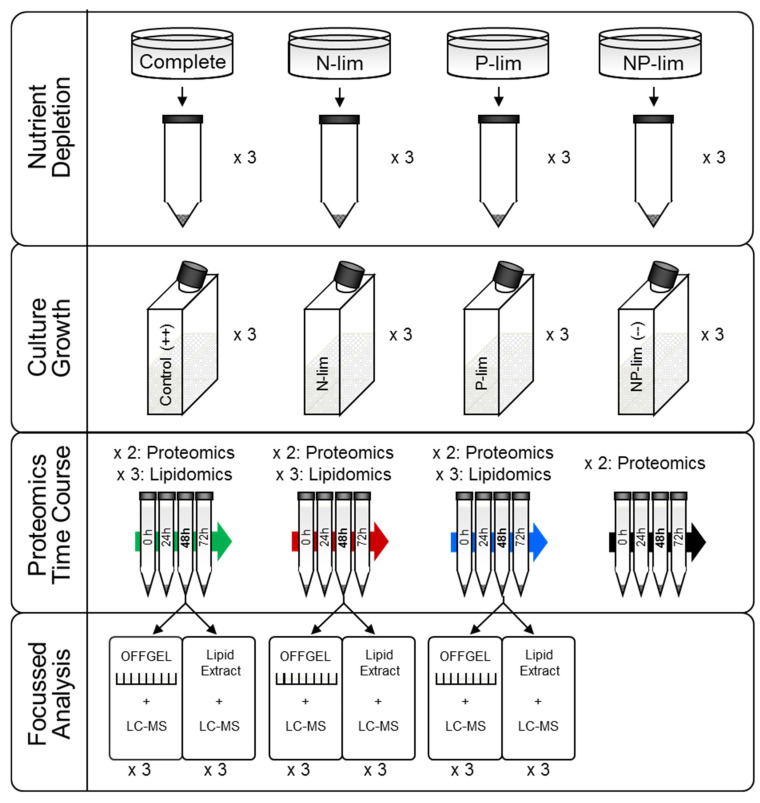
Experimental workflow for proteomic and lipidomic profiling of *O. tauri* under nutrient stress. Cultures were grown in complete media, media lacking nitrogen sources (N-dep), media lacking phosphorus (P-dep) or media lacking both nitrogen and phosphorous (NP-dep). Algae were sampled at *t* = 0, 24, 48 and 72 h and shotgun proteomics was performed. The tryptic digests from the 48 h time point were additionally fractionated using OFFGEL isoelectric focusing (IEF) to permit more in-depth characterisation of the *O. tauri* proteome. Algal samples at this time point were also subjected to lipidomic analysis in order to investigate changes in lipid metabolism.

Wild type *O. tauri* were cultured in 12 identical flasks of 200 mL artificial sea water (Instant Ocean 30 ppt) supplemented with ammonia (NH_4_Cl 36 µM), nitrate (NaNO_3_ 880 µM), phosphate (β-glycerophosphate 10 µM) and also silica, selenium, Keller metals, vitamins and antibiotics for 7 d in 12/12 blue light/dark cycles at 20 °C as described previously [53]. On day 7, cells were centrifuged in 50 mL tubes (10 min at 3200× *g*). Pellets were washed twice by resuspension in 10 mL of the respective new media, before centrifuging again and resuspending pellets together in triplicates of 200 mL of either complete media, media lacking nitrogen (N-dep), media lacking phosphate (P-dep) or media lacking both nitrogen and phosphate (NP-dep). Culture growth in the three replicates of each condition was monitored by optical density and related to cell numbers using previously acquired FACS data. 1 mL was removed from each culture and concentrated by centrifuging (5 min at 10,000× *g*) and resuspending in 50 µL. Absorbance was measured at 600 nm using a NanoDrop spectrophotometer (Thermo Scientific, Hemel Hempstead, UK). 20 mL of each of the 12 cultures was sampled at midday every 24 h for up to 72 h (4 time points). A double quantity was harvested at *t* = 48 h for sample peptide fractionation and for lipid analysis. Final pellets were resuspended in 2 mL PBS before centrifuging again to wash. Washed pellets were lysed in 400 µL dH_2_O before freezing. The overall experimental design and naming convention is shown in Figure 6.

### 3.2. In-Solution Trypsin Digestion

Samples were centrifuged (10 min at 3200× *g*) and resuspended in 500 µL of ice-cold PBS before centrifuging again. Pellets were lysed in 125 µL 8 M urea and the protein concentration of each culture sample was determined using the Bradford Assay (Thermo Scientific, Loughborough, UK). The protein samples (approximately 50 µg) were diluted in water to 300 μL. The proteins were reduced by incubating with 125 μL 8M urea, 25 μL 1 M ammonium bicarbonate and 25 μL 200 mM DTT for 30 min at room temperature. This was followed by cysteine alkylation with 25 μL of 500 mM iodoacetamide for 45 min at room temperature in the dark. Trypsin (Roche, Lewes, UK) (10 µg) was added and the digestion was allowed to proceed overnight at 37 °C. The digestion was terminated by addition of formic acid. 

### 3.3. Fractionation of Peptides

Desalted peptides from the 48 h time point were fractionated by isoelectric focussing (IEF) on an OFFGEL 3100 system (Agilent, Stockport, UK). Samples (approximately 50 µg of protein) were incubated at room temperature for 1 h and then applied to IPG strips (pH 3–8, 13 cm, GE Healthcare, Amersham, UK), which were run until 20 kVh were reached with a voltage maximum of 4.5 kV and a current limit of 50 µA. The eight peptide fractions were subsequently desalted using Bond Elut OMIX C18 (Agilent, Stockport, UK).

### 3.4. Protein Identification and Data Analysis

Peptides were analysed by capillary LC-MS/MS using a Thermo LTQ-Orbitrap XL mass spectrometer coupled to an Agilent 1200 binary HPLC system using the method of Le Bihan et al. [54]. Peptide identification was performed using Mascot (version 2.3, Matrix Sciences, London, UK) and the *O. tauri* subset of the NCBI protein database. The initial search parameters allowed for two trypsin missed cleavages, carbamidomethyl modification of cysteine residues, oxidation of methionine, acetylation of N-terminal peptides, a precursor mass tolerance of 7 ppm and a fragment mass tolerance of ±0.4 Da. Only proteins identified by two or more peptides and a Mascot score larger than 20 were retained. Data from these eight fractions were pooled and analysed using a label-free quantitation analysis in Progenesis LC-MS (version 4.0, Nonlinear Dynamics, Newcastle, UK) to determine protein intensities and statistically significant differences (ANOVA, *p* ≤ 0.05, fold change ≥1.5) between the complete media, N-dep, P-dep and NP-dep study groups. Protein co-expression analysis was performed in a similar manner as in Kanonidis et al. [55] on arcsinh transformed data. Gene Ontology term enrichment was done using topGO [34].

### 3.5. Lipid Extraction

The algal lipids were extracted according to the method of Folch et al. [56]. Briefly, a 150 µL aliquot was extracted with 3 mL chloroform/methanol (2/1, *v*/*v*). The mixture was then left to stand at room temperature for 1 h. The samples were partitioned by the addition of 600 µL of 0.1 M KCl and the mixture was centrifuged to facilitate phase separation. The lower chloroform layer was evaporated to dryness under nitrogen gas, reconstituted in 500 µL methanol/chloroform (2/1, *v*/*v*) and diluted 1:20 with methanol. 

### 3.6. Analysis of Lipids and Data Processing

Global lipidomic analyses were undertaken on a Thermo Exactive Orbitrap platform over m/z 200–2000 in positive and negative ion modes. The mass spectrometer was interfaced to a Thermo Accela 1250 UHPLC system (Hemel Hempstead, UK). Algal lipid extracts were loaded on to a Thermo Hypersil Gold C18 column (1.9 µm; 2.1 mm × 50 mm) over a 21 min gradient starting at 65% Buffer A (10 mM ammonium formate and 0.1% (*v*/*v*) formic acid) and 35% Buffer B (90:10 isopropanol/acetonitrile with 10 mM ammonium formate and 0.1% (*v*/*v*) formic acid) increasing to 100% of Buffer B. Progenesis QI (Nonlinear Dynamics) was used to process the datasets. Lipids were identified through interrogation of HMDB (http://www.hmdb.ca/), LIPID MAPS (www.lipidmaps.org/) and a locally generated algal lipid database. Multivariate statistical analysis was performed using SIMCA-P v13.0 (Umetrics, Umea, Sweden). The processed data were transformed using variance stabilisation and the mean abundance within experimental conditions was determined. Lipids were deemed to be altered in abundance following a two-way ANOVA analysis with fold-change differences between groups determined.

## 4. Conclusions

Algal metabolism comprises complex networks and, as such, integrated approaches are required to investigate the effects of fluctuating nutrient availability at the molecular level. Depriving *O. tauri* of nitrogen and/or phosphorus resulted in several specific adaptations, demonstrating that the limitation of nutrients is sensed and compensated for as a survival strategy. Proteomic analysis revealed that, in the absence of external nitrogen, there were higher levels of expression of ammonia and urea transporters, whilst phosphate depletion led to increased expression of phosphokinases and phosphate transporters, suggesting an attempt to maximise scavenging opportunities as opposed to energy conservation. In both nutrient-limiting conditions, enzymes involved in stress starch synthesis were increased and ribosomal and nitrate transport proteins were reduced. Despite these findings, a significant proportion of identified proteins were of unknown function (in an otherwise well-annotated genome), pointing towards further work on a number of mechanisms in *O. tauri* that are distinct from sequenced and studied species. Our lipidomic methodology also provided the sensitivity and capacity required for the rapid analysis of lipid populations in *O. tauri.* The changes in lipid profiles reflected the nutrient stress with nitrogen deprivation, leading to the accumulation of TG, a well-characterised response in algae. Furthermore, under phosphorus-limiting conditions *O. tauri* adjusted its metabolism towards the production of betaine lipids, a finding that was reinforced by the putative identification of a protein containing domains homologous to BTA1. Our work extends previous studies focused on lipid analysis of *O. tauri* and provides additional novel insights at the protein level into the adaptive processes of the smallest known free-living eukaryote to changing environmental conditions.

## Supplementary Materials

The following are available online at https://www.mdpi.com/2218-1989/10/7/273/s1, Figure S1: Phylogenetic analysis of key proteins involved in nutrient deprivation. Figure S2: Representative negative ion LC-MS chromatograms of lipid profiles of *O. tauri* cultured in nutrient-limiting conditions. Figure S3: PCA scores plots of algal lipid profiles. Lipidomic data sets generated in (A) positive and (B) negative ion modes. Figure S4: OPLS-DA scores plots and associated S-plot of negative ion mode lipidomics data. Table S1: Label-free shotgun proteomic analysis of *O. tauri* under nutrient stress. Table S2: Differential expression of proteins in focussed proteomic analysis, Table S3: Putative identifications of altered lipids in *O. tauri* (positive ion mode). Table S4: Putative identifications of altered lipids in *O. tauri* (negative ion mode).
