# Supplementary material for: Surviving Starvation: Proteomic and Lipidomic Profiling of Nutrient Deprivation in the Smallest Known Free-Living Eukaryote
Source: Metabolites. 2020 Jul 3;10(7):273. doi: 10.3390/metabo10070273 (PMC7407893; doi:10.3390/metabo10070273)
Supplement: Supplementary file 1 [file metabolites-10-00273-s001.zip › metabolites-776545-sup for author-updated/Figures.docx]

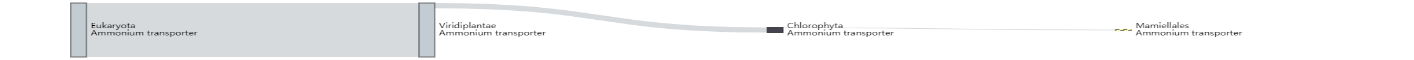

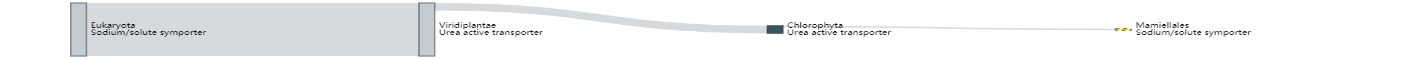

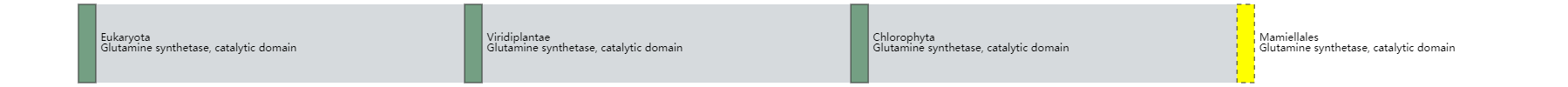

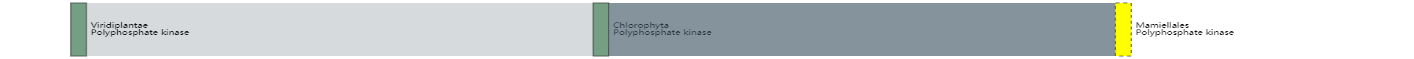

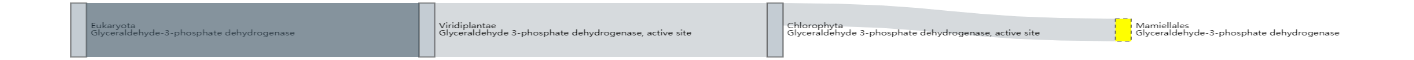

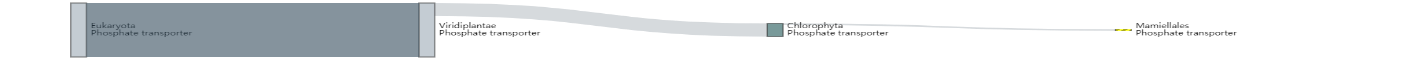


**Figure S1.** Phylogenetic analysis of key proteins involved in nutrient deprivation.


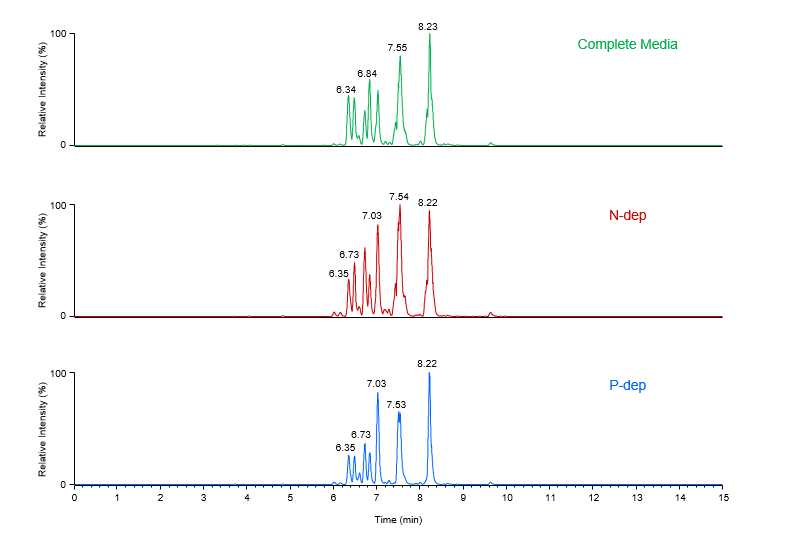


**Figure S2.** Representative negative ion LC-MS chromatograms of lipid profiles of *O. tauri* cultured in nutrient-limiting conditions. Algal lipids were analysed by high-resolution LC-MS.


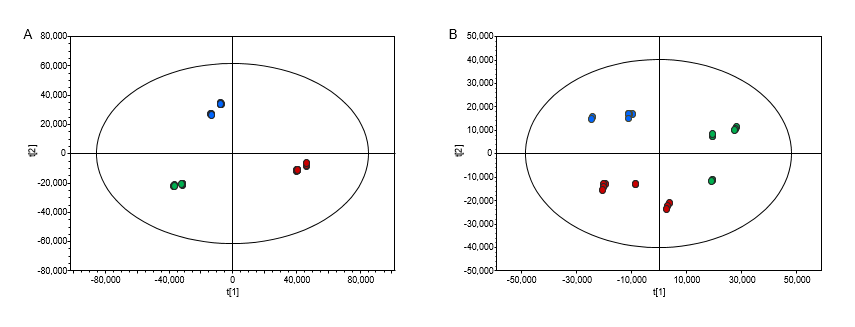


**Figure S3.** PCA scores plots of algal lipid profiles. Lipidomic data sets generated in (A) positive and (B) negative ion modes. Key: Complete media = green; N-dep = red; P-dep = blue.


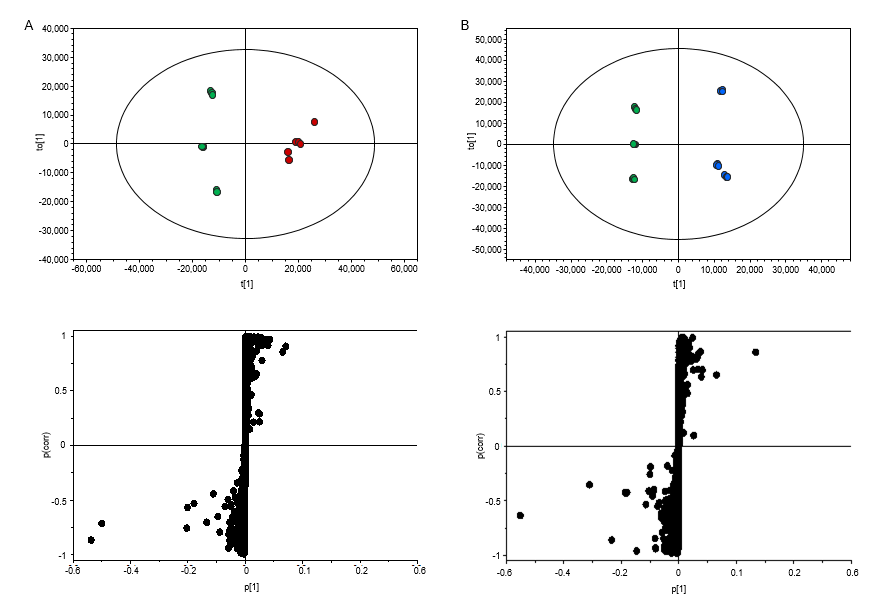


**Figure S4.** OPLS-DA scores plots and associated S-plot of negative ion mode lipidomics data. Conditions: (A) N-dep conditions and (B) P-dep conditions.
